# Preparation and Optimization of Voriconazole Gel 0.25% for Topical Delivery in Terbinafine Resistance Dermatophytosis: In Vitro and Pilot Animal Study

**DOI:** 10.1155/drp/5995621

**Published:** 2026-04-21

**Authors:** Saman Ahmad Nasrollahi, Mahsa Fattahi, Ensieh Lotfali, Atefeh Naeimifar, Fatemeh Amiri, Aliasghar Ghaderi, Pegah Tamimi, Shayan Zamani, Ali Khamesipoor, Alireza Firooz

**Affiliations:** ^1^ Center for Research and Training in Skin Diseases and Leprosy, Tehran University of Medical Sciences, Tehran, Iran, tums.ac.ir; ^2^ Immunology, Asthma and Allergy Research Institute, Tehran University of Medical Sciences, Tehran, Iran, tums.ac.ir; ^3^ Children’s Medical Center, Pediatric Center of Excellence, Tehran University of Medical Sciences, Tehran, Iran, tums.ac.ir; ^4^ Department of Medical Parasitology and Mycology, School of Medicine, Shahid Beheshti University of Medical Sciences, Tehran, Iran, sbmu.ac.ir; ^5^ Department of Pharmaceutics, Faculty of Pharmacy, Tehran University of Medical Sciences, Tehran, Iran, tums.ac.ir; ^6^ School of Medicine, Tehran University of Medical Sciences, Tehran, Iran, tums.ac.ir

**Keywords:** antifungal treatment, severe dermatophytosis, topical delivery, voriconazole gel 0.25%

## Abstract

**Background:**

There have been several reports of patients with chronic recalcitrant diseases, rare presentations, recurrent relapses, and treatment failure. This study aimed to formulate and evaluate the safety profile of topical voriconazole (VCZ) gel 0.25% for effective drug delivery to the skin as an adjuvant therapy in cases of extensive dermatophytosis.

**Methods and Materials:**

VCZ 0.25% topical gel was manufactured by Carbomer, and its characteristics such as pH, viscosity, density, and microbial growth were examined at 40 ± 2°C/75 ± 5% RH for six months. The animals were divided into three groups (2:2:2) randomly. The hair from the back of guinea pigs (test sites: 2 cm^2^) was shaven, and their skin was scraped slightly using a single‐use scalpel. Afterward, terbinafine drug–resistant *Arthroderma benhamiae* suspension (50 μL) was inoculated to a 2 cm^2^ surface area of the shaven skin.

**Results:**

Agar culture of scrapings from the area indicated the infection development. The fabricated VCZ topical gel exhibited acceptable stability. The VCZ 0.25% gel was proved to be an effective treatment in a time‐dependent base, in comparison with terbinafine (1%) as a positive control (*p* < 0.05). Furthermore, using gel base VCZ 0.25% reduced the period of full treatment of *A. benhamiae* infection to five days, compared with the 12‐day complete cure by terbinafine.

**Conclusion:**

Results from the present study indicated that gel‐based VCZ could be utilized as topical therapy for dermatophytosis. Due to the increase of drug‐resistant dermatophyte spp., indications of gel base VCZ 0.25% for treating dermatophytosis are believed to increase accordingly.

## 1. Introduction

Dermatophytosis, especially tinea corporis, is a prevalent superficial skin infection [1]. A survey on the prevalence of outpatient dermatologic chief complaints demonstrated that 20%–25% of those who present for clinical consults suffer from fungal skin infections globally [2]. With the increase in international travelers, dermatophytosis is no longer limited to certain geographic regions. There have been several reports on patients with chronic recalcitrant diseases, rare presentations, recurrent relapses, and treatment failure [3–7].

The emergence of terbinafine resistance has been reported in Denmark [8], Switzerland [9, 10], China, Belgium [11], Germany [12], Japan [13], Iran [14, 15], Finland [16], and Bahrain [17]. There is a pressing demand for the development of new antifungal agents, new formulations to enhance drug delivery, novel molecular targets in fungal pathogens, and remodeling existing drugs.

In the recent trials, oral voriconazole (VCZ) was found to have superior effectiveness in the case of dermatophyte strains which showed resistance to terbinafine and azoles. Reports also mention that it improved the duration of complete cure without recurrence. Since then, physicians have begun to increasingly prescribe it for drug‐resistant cases [7].

VCZ is a triazole antifungal structurally similar to fluconazole while having a broad spectrum activity resembling itraconazole [18]. VCZ was first approved by the Food and Drug Administration (FDA) for the treatment of invasive aspergillosis and refractory illnesses of *Scedosporium apiospermum* and *Fusarium* spp. It has been proven to be effective in treating febrile neutropenia [19]. Several side effects have been attributed to VCZ including photophobia, hallucinations, thrombocytopenia, and acute renal failure [20]. In the present study, to minimize the intensity and frequency of the associated side effects, the formulation was developed for the delivery of VCZ to the topical site. As opposed to creams and ointments, gels facilitate the release of drug substances, irrespective of the water solubility of the drug. Gels are also associated with a lower inflammation risk and adverse reactions, are easy to apply, and do not need removal. A gel base formulation of fluconazole considered for topical delivery of fluconazole proved to be effective due to easy preparation, stability, safety, and cost‐effectiveness [21].

For a long time, the imidazole antifungal agent clotrimazole has been topically used to treat superficial dermatophytosis.

This study aimed to formulate this medication and evaluate the safety and effectiveness of topical VCZ gel 0.25% for effective drug delivery to the skin in cases of cutaneous dermatophytosis.

## 2. Materials and Methods

### 2.1. Preparation’s Ingredients

VCZ powder was provided by Sigma‐Aldrich (Taufkirchen, Germany). Propylene glycol and glycerin were obtained from Sepidaj Company (Tehran, Iran). Carbomer was obtained from Chimica Pomponesco Spa Co. (Italy). Methylparaben and propylparaben were purchased from Alborz Bulk (Saveh, Iran). Soybean Casein Digest Broth (Trypticase Soy Broth [TSB]), Trypticase Soy Agar (TSA), Sabouraud Dextrose Agar (SDA), Mannitol Salt, and Cetrimide Agar were acquired from Liofilchem (Roseto, Italy). The dialysis membrane (MEMBRA‐CEL MD44) with a molecular weight cutoff value of 12,000–14000 Da was from Sigma‐Aldrich (Taufkirchen, Germany). Deionized water was prepared whenever necessary (Millipore, Bedford, MA).

### 2.2. Preparation of the Topical Gel

Methylparaben and propylparaben were first dissolved in distilled water at 90°C using a hot plate under continuous stirring. After cooling the solution to 30°C, propylene glycol, glycerin, and carbomer were gradually added and mixed until complete dissolution and uniform dispersion were achieved. VCZ was then incorporated into the formulation and homogenized at 25 ± 1°C with a stirring speed of 1200 rpm for 20 min. Finally, the pH of the formulation was adjusted to 4.6 using 1 M sodium hydroxide (NaOH), resulting in the formation of a homogeneous and viscous gel. The final VCZ concentration in the gel was 0.25%.

### 2.3. Physicochemical Characterization

#### 2.3.1. Stability Study

Accelerated stability analyses (i.e., appearance, color, and odor) were conducted at 40 ± 2°C/75 ± 5% relative humidity (RH) for six months following International Council for Harmonization (ICH) guidelines [22].

#### 2.3.2. Determination of pH, Density, and Viscosity

A digital pH meter (Metrohm 827‐ Switzerland) was used to measure the pH of the preparation.

The precise volume of the pycnometer was determined by filling it with water to measure the density. The container was weighted to measure the density with the following formula:

*p* = mV.

Here, *p* = density, *m* = mass, and V = volume.

A Polyvisc Viscometer (Brookfield‐USA) was used to measure the gel viscosity. The measurements were conducted at 25°C, in triplicate.

#### 2.3.3. Drug Content and Uniformity

The uniformity of VCZ distribution within the gel was assessed by HPLC (or UV spectrophotometry), confirming content within ±5% of the target concentration.

### 2.4. Microbial Assessments

To rule out bacterial contamination of the gel, two dilutions (10^−1^,10^−2^) were prepared: 10‐mL pure liquid of VCZ gel 0.25% was diluted in 90‐mL phosphate buffer (pH = 7.2) to prepare the 10^−1^ concentration, and 10 mL of 10^−1^ solution was diluted with another 90‐mL phosphate buffer (pH = 7.2) to 10^−2^ concentration. Hundred μL of each of 10^−1^ and 10^−2^ solutions were inoculated on two media of TSB culture medium (Merck, Germany) and TSA culture medium (Merck, Germany); 4 plates in total. Plates were then incubated for 48 h at 35°C.

Fungal contamination of the preparation can also be a major confounding factor in the analysis. To rule out this possibility, 100 μL of each of 10^−1^ and 10^−2^ solutions were inoculated were transferred to 2 plates of SDA (Merck, Germany) and incubated at 35°C for 48 h. Colony counting was performed. Counting was passed in plates containing less than 100 colonies for microbial counting (TAMC) and fewer than 10 colonies for fungal counting (TYMC) [23].

### 2.5. In Vitro Antifungal Activity of VCZ Gel 0.25%

#### 2.5.1. Agar Well Method

##### 2.5.1.1. Antifungal Serial Dilution Preparation

For evaluation of the antifungal susceptibility of strains to VCZ, first, two forms of VCZ solutions were prepared, VCZ gel 0.25% and a working solution. The working solution was made in this manner: first, a VCZ solution containing 64 μg/mL was prepared using VCZ powder (Sigma‐Aldrich, USA). Thereafter, a diluted solution containing 2 μg/mL (equivalent to VCZ 0.2%) was achieved using the serial dilution method [24]. Another working solution was made with amphotericin B powder with the same concentration as VCZ gel and working solution (2 μg/mL), which was used as the positive control. Amphotericin B was chosen as the reference antifungal based on its broad‐spectrum activity and established efficacy in experimental models. This selection was further supported by the reduced susceptibility of the study isolate to commonly used antifungal agents.

##### 2.5.1.2. Preparation of Fungal Inoculums

To evaluate the drug’s efficacy against fungi in the agar well method, 8 archived fungal isolates were chosen. These included 5 strains of *Trichophyton indotineae* (which showed resistance to terbinafine, itraconazole, and fluconazole [7] in the antifungal susceptibility testing based on standards of Clinical and Laboratory Standards Institute [CLSI]) [24] and 1 strain of *Arthroderma benhamiae* (resistance to terbinafine), 1 *Trichophyton rubrum* (which showed resistance to terbinafine, itraconazole, and fluconazole), and 1 *Microsporum canis* (sensitive to all antifungal agents). Members of the *T. rubrum*, *T. mentagrophytes*, and *M. canis* complexes have become the major species of urban populations globally [25]. *Trichophyton rubrum* and *M. canis* generally causes mild skin infections. *T. indotineae* cause the most cases of severe recalcitrant ringworm. All strains were obtained from clinical samples and archived in our fungi bank. It is important to mention that the resistant isolates also showed clinical resistance, defined as lack of improvement or persistence of infection in patients despite standard antifungal therapy.

*T. indotineae* and *A*. *benhamiae* were identified using culture followed by internal transcript spacer (ITS1‐2 region) sequencing. Prior to this study, a large number of species, including the *T. mentagrophytes* complex, were successfully resolved. As diagnostic parameters with a high degree of predictivity, ITS sequencing as yet is recommended [26, 27].

To harvest the fungal spores, 20 μL of archived TSB suspensions of fungal strains were streaked on 6 plates of Mycosel agar (Merck, Germany). The plates were then incubated for 14 days at 29 ± 2°C for fungal growth. To prepare the fungal suspension for the upcoming agar well test, 1 mL of physiologic serum and 20 μL of Tween 20% solution ((Merck, Germany) were added to the surface of each of the harvested agar plates. For each of the plates, 1 mL of the obtained solution on the agar surface (containing the fungal spores) was transferred to a cryovial and kept as a stock for the upcoming experiments. Stocks were placed in a vortex blender to detach the fungal spores and then were set aside for 15 min at room temperature to allow the heavier particles to precipitate. The upper homogenous parts of the attained suspensions were transferred to another cryovial each, for the next step. Five sterile falcon tubes were prepared, each containing 5 mL of physiologic serum. Then, 100 μL of each was removed and replaced by 100 μL of the fungal spore suspensions. The prepared suspensions were then adjusted with a sterile physiologic solution to match the opacity of 0.5 McFarland’s standard.

#### 2.5.2. Agar Well Step [28]

To perform an antifungal susceptibility assay by the diffusion streak approach, the agar‐based medium was aliquoted in 24 sterile Petri dishes and was set aside to solidify. Twenty μL of each of the fungal suspensions (*T*. *indotineae*, *A*. *benhamiae*, *T*. *rubrum*, and *M*. *canis*) were streaked over the surface of the prepared agar medium (4 plates for each of the strains). The reason for choosing and implementing different species was to investigate the fungicidal or fungistatic properties of VCZ on the species. Then, a well was created in each of the agar media using a sterile Pasteur pipette. Subsequently, 20 μL of each of the VCZ working solution 0.2%, VCZ gel 0.25%, amphotericin 0.2%, and distilled water were added to the wells on separate plates. Distilled water served as a negative control, while amphotericin B was the positive control. Then, the plates were incubated at 28°C for 48–72 h; inhibition zone diameters were determined following the incubation using an elliptical ruler. It repeated 3 times for each of the strains.

### 2.6. Animal Model

#### 2.6.1. Ethical Approval

This study has been approved by the Ethics Committee of Tehran University of Medical Sciences (IR.TUMS.MEDICINE.REC.1399.1071).

#### 2.6.2. Animals

Guinea pig was chosen as our animal study model due to its most similar skin structure to human skin. Six brown male guinea pigs, aged 7 weeks, with a weight of 300 ± 10 gr, were kept in the animal house and permitted to acclimate to the new environment for at least seven days. These pigs were fed with pelleted food and water in rooms at 20°C–22°C and 70% humidity in a 12:12 light–dark cycle.

#### 2.6.3. Tested Isolates for the Animal Model

*A. benhamiae* is easy to adapt to guinea pigs because it is zoophilic, so it was chosen as the infectious pathogen for the animal model. Compared with *M*. *Canis*, this species can adapt better to guinea pig and develop more inflamed lesions. This isolate showed resistance to terbinafine in the clinic. Laboratory resistance was confirmed by in vitro tests, and mutation in the squalene gene was detected.

#### 2.6.4. Study Design

The hair on the midback of guinea pigs (surface: 2 cm^2^) was shaved, and their skin was scraped slightly using a single‐use scalpel. Afterward, 50 μL of *A*. *benhamiae* suspension (0.5 McFarland unit) was inoculated into the 2 cm^2^ shaved area. There was a pause and follow‐up of 14 days for the infection to present itself on the back of the guinea pigs. The samples were then randomly divided into three groups (2:2:2). Randomization was conducted using a computer‐generated random sequence by an investigator who was not involved in treatment administration, clinical evaluation, or data analysis, in order to minimize allocation bias.

Blinding was implemented at the level of outcome assessment.

• **Group I** was treated topically with VCZ gel 0.25%, twice daily (1 mL/cm^2^) for a maximum of 30 days. If the guinea pigs were treated sooner than this, the treatment was discontinued.
• **Group II** (positive control) was treated topically with terbinafine cream 1% (Behvazan, Iran) (1 mL/cm^2^), twice daily, for a maximum of 30 days. If the guinea pigs were treated sooner than this, the treatment was discontinued.
 Terbinafine 1% was selected as the standard topical comparator, reflecting commonly used clinical formulations. Although the concentrations of the test and comparator treatments differ, the study was designed to compare relative therapeutic outcomes using established clinical dosing regimens rather than equimolar potency. This approach ensures that the results are relevant to commonly applied clinical practice and provides a meaningful comparison of treatment efficacy.
• **Group III** (negative control) received no treatment during the study. Following the completion of our research period, every animal was given the same treatment as Group II and subsequently sent back to the animal house of the laboratory. None of the animals were subjected to euthanasia.

Lesions for each of the groups were sized as days 0, 7, 10, 14, 21, and 30. On each of these endpoints, the lesion healing rate (LHR) was calculated by the following formula: LHR (%) = [(Lo‐Lu)/Lo] × 100 (Lo: original lesion area and Lu: unhealed lesion area) and registered.

In case the lesions in the VCZ gel 0.25% group were resolved, the efficacy of the drug was double‐evaluated via performing culture from skin scales of the area on SDA (Merck, Germany) to screen the presence of viable *A. benhamiae*. The incubation of cultures was performed at 28°C for 2 weeks. In case the lesions faded, the guinea pigs were followed daily for 30 days, to rule out relapse.

### 2.7. Statistical analysis

All data were analyzed by software IBM SPSS Version 29. The data were analyzed by Fisher’s exact test. Significant was defined as *p* value < 0.05.

## 3. Results

### 3.1. Estimation of Stability, pH, Density, and Viscosity of VCZ Topical Gel 0.25%

The fabricated 0.25% VCZ topical gel exhibited acceptable organoleptic characteristics and maintained physical stability throughout the study period. Accelerated stability testing was performed according to ICH guidelines under conditions of 40 ± 2°C and 75 ± 5% RH for six months. No appreciable changes in appearance, color, or odor were detected, confirming the stability of the formulation (Table 1).

**TABLE 1:** Results of stability assessment and antimicrobial test of voriconazole gel 0.25% at 40 ± 2°C and the 75 ± 5% RH for 6 months.

Test	Period of storage (40°C ± 2°C, 75% RH ± 5% RH)			
	Initial	3^rd^ M	6^th^ M	Standards
Description	Complies	Complies	Complies	Transparent and homogenous gel
Odor	Complies	Complies	Complies	Characteristic chemical order
pH	4.51	4.60	4.54	NLT 4.50 NMT 7.50
Viscosity (cP) at 25°c	1113	1070	1123	NLT 1000 NMT 3000
Density (g/cm3)	0.976	0.981	0.98	NLT 0.999 NMT 1.0300
Total bacterial count (CFU)	5	1	2	NMT 100
Total fungi and yeast count (CFU)	7	0	1	NMT 10
*P. aeruginosa*	Negative	Negative	Negative	Negative
*S. aureus*	Negative	Negative	Negative	Negative

Abbreviations: NLT, not less than; NMT, not more than.

The mean pH was 4.51, 4.60, and 4.54 at the initial, third, and sixth months, respectively. Moreover, the pH values were the same as the normal pH values of the skin. There was no significant difference in pH values during the preparation and after the six months. The viscosity was 1113, 1070, and 1123 CPA at the initial, third, and sixth months. The density of fresh gel was 0.976 and was calculated at 0.971 and 0.980 in the third and sixth months.

The microbial evaluation of the whole stability period showed that the colonies and fungal counts were less than 10, representing that antimicrobial activity has been acceptable and effective. Preservatives were in the desired range of antimicrobial activity.

### 3.2. In Vitro, Antifungal Activity of VCZ Gel 0.2% Formulation Using the Agar Well Approach

Antifungal activity of VCZ in a gel formulation was tested against test fungi through the agar well method to confirm the fungistatic activity in all drug resistance and sensitive species.

The verification test of resistance of all isolates to terbinafine indicated that all 5 *T. indotineae* strains and *T. rubrum* were once again read as resistant (MIC = 2 μg/ML).

Criteria cutoff values for inhabitation zone diameters (IZDs) for VCZ 0.2% and VCZ 0.25% gel are defined based on Agarwal experience with some modification for classifying the isolates as resistant, intermediate, or sensitive as shown in Table 2 [28] and Table 3.

**TABLE 2:** Cutoff values for inhabitation zone diameters for tested antifungals.

Antifungal agents	Mean ± SD	Sensitive Mean ‐1SD	Tolerant Mean ‐1SD to mean ‐2SD	Resistant < Mean ‐2SD
VCZ 0.2%	41.5 ± 5.6	> 36	30–36	30 >
VCZ gel 0.25%	43.5 ± 5.6	> 38	32–38	32 >
AMB	49.5 ± 0.8	> 48	47–48	47 >

*Note:* VCZ: voriconazole; AMB: amphotericin B.

**TABLE 3:** Antifungal susceptibility pattern of *T. indotineae* and *T. rubrum* to three agents based on criteria from this study.

Antifungals	Total IZD (mm)	Average IZD (mm)	S/R
VCZ 0.2%	*T. indotineae*: 249 *T. rubrum*: 247	*T. indotineae*: 41.5 *T. rubrum*: 40	S

VCZ gel 0.25%	*T. indotineae*: 261 *T. rubrum*: 260	*T. indotineae*: 43.5 *T. rubrum*: 42	S

AMB	*T. indotineae*: 269 *T. rubrum*: 270	*T. indotineae*: 44.8 *T. rubrum*: 45	S

*Note:* VCZ: voriconazole; AMB: amphotericin B; S: sensitive; R: resistant.

Abbreviations: IZD, inhibition zone diameters; MIC, minimum inhibitory concentration.

**FIGURE 1:**
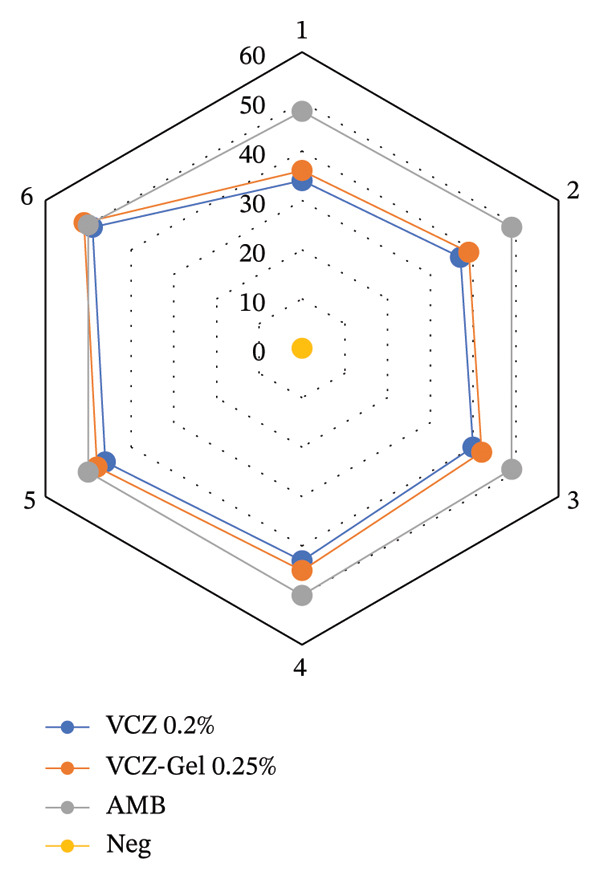
The inhibition zones of voriconazole gel 0.25%, voriconazole 0.2%, and Amphotericin B against dermatophyte species, as shown in the radar chart, are as follows: *T. indotineae* (*n* = 1−4), *T. rubrum* (*n* = 5), and *A. benhamiae*, and *M. canis* (*n* = 6) showed similar results. The differences were not statistically significant *p* < 0.05.

By comparing the radar charts, we found that no significant differences were seen for the efficacy of VCZ gel 0.25% against the pathogenic dermatophytes when compared with positive indicators (*p* = 0.05) (Figure 1), while it had higher activity than VCZ 0.2% (*p* = 0.04), which indicated the stronger antidermatophyte activities of VCZ gel 0.25%. No zone was developed in the negative control.

### 3.3. In Vitro, Antifungal Activity of VCZ Gel 0.25% Formulation Using the Broth Dilution Method

To ensure the accuracy of antifungal results and to determine if resistance to VCZ could develop, a minimum inhibitory concentration was designed to detect the change in sensitivity after successive passages of tested organisms. In contrast, a progressive increase in resistance to nystatin was observed.

### 3.4. In Vivo Antidermatophytic Activity of VCZ Gel

Finally, we compared the in vivo antifungal activity of VCZ gel 0.25% and topical terbinafine 1% using the animal dermatophytosis model. Table 3 shows the LHR in the groups. The ratios of lesion areas to the initial lesion area on days 0, 7, 10, and 30 were calculated.

In the VCZ gel, 0.25% topical treatment group healing started 3 days after the initiation of treatment. At 7 days, the lesion of the VCZ gel 0.25% topical‐treated guinea pig no. 1 had integrated into the surrounding skin, and their color was similar to normal skin, and in the untreated groups, no integration into the lesion was seen. At 10 days, the VCZ gel 0.25% topically treated guinea pig no. 2 exhibited significantly accelerated obvious lesion healing compared with terbinafine 1% and untreated groups (*p* < 0.005).

In the terbinafine, 1% topical treatment group healing started 3 days after the initiation of treatment. At 3 days posttreatment, treatment and positive control groups showed a similar pattern with minimal lesion healing evident. The terbinafine 1%‐treated group on days 7 and 10 showed a minor to moderate increase in the regeneration of the epiderm. At 30 days, successful lesion closure was attained for the terbinafine 1%‐treated group with LHR: 90%.

Safety was monitored throughout the study period by regular clinical observation of all animals. Evaluations included assessment of skin condition at the application site, behavioral changes, and general health status. No signs of local skin irritation, inflammation, or ulceration were observed in any of the treated groups. In addition, no systemic adverse effects or abnormal behaviors suggestive of toxicity were detected during the treatment period. Overall, the topical formulations were well tolerated under the experimental conditions. The relapses of dermatophytosis in guinea pigs were assessed for 30 days after complete cure, and no relapse was observed.

## 4. Discussion

The gel base VCZ had no impact on the skin membrane of animals owing to the existence of emollient properties induced by the gelling constituent, carbomer. The antifungal analysis of optimized formulation against *T. indotineae* strains and *T. rubrum* yielded satisfactory results with the inhibition diameter zone, mean ± SD: 43.5 ± 5.6. Gel base VCZ has maximum fungistatic efficacy, owing to substantial penetration into fungal cell membranes and inhibition of CYP450‐dependent 14‐a sterol demethylase as well as ergosterol synthesis in the cell wall of fungi.

To deliver the drug to the active site, the dermatologist and pharmaceutics keep increasing the dose of the drug in the topical formulations. The topical terbinafine 1% or topical clotrimazole was the most topical antifungal agent used alone or in combination with oral antifungal healing to cure dermatophytosis. The spread in drug resistance species raises the hypothesis that the dose of antifungal was increased and may initiate emerging resistance in dermatophyte species and an increase in the unfavorable effects due to long‐continued therapy. The capacity of the drug to be delivered by a vehicle and infiltrate the skin at sufficient amounts and adequate rates is essential for effective topical healing.

Terbinafine topical formulation was tested as a control. Both compounds were utilized twice daily corresponding to the clinical application. The efficacy of gel base VCZ 0.25% after application indicated that it could quickly reach the target site in comparison to terbinafine 1% (*p* = 0.05).

Across the reviewed studies, topical formulations of VCZ consistently demonstrated advantages over systemic therapy and conventional topical antifungals, particularly in terms of localized efficacy, skin penetration, and safety.

Conventional topical VCZ cream and gel formulations were shown to be effective alternatives when systemic VCZ use was limited by hepatotoxicity, neurotoxicity, or drug–drug interactions. A 1% VCZ cream remained stable for up to 90 days under refrigerated conditions and achieved complete clinical resolution of cutaneous *Fusarium solani* infection after failure of oral VCZ therapy. Similarly, carbomer‐based VCZ gels exhibited good skin compatibility, attributed to the emollient nature of the gelling agent, and did not damage the animal skin membrane. These formulations showed strong antifungal activity against *T. indotineae* and *T. rubrum*, with a large mean inhibition zone (43.5 ± 5.6 mm), confirming potent fungistatic effects mediated by inhibition of CYP450‐dependent 14‐α‐sterol demethylase and ergosterol synthesis [29].

More advanced nanotechnology‐based VCZ delivery systems further improved antifungal performance. VCZ‐loaded microemulsion‐based hydrogels (MBHs) exhibited broad‐spectrum antifungal activity against yeasts and molds, including *Saccharomyces*, *Rhizopus*, *Actinomyces*, and *Candida* species. Among these, optimized formulations demonstrated superior inhibition profiles compared with nonoptimized systems [30]. Heyon et al. assessed nanostructured lipid carrier‐based hydrogel (NLC‐gel) formulation to determine skin permeation as well as retention profile in vitro [31]. In another study on skin permeation by a Franz diffusion cell mounted with depilated mouse skin, the NLC‐gel proved to be more effective than regular formulations of cream and microemulsion‐based gel, with 2.8‐ and 1.7‐fold higher flux values, respectively. Furthermore, the NLC‐gel caused a higher collection of VCZ in deeper layers of skin compared with reference formulations [32]. The most recent study introduced an innovative VCZ nanoemulsion combined with Pinus sylvestris essential oil (PSEO). This formulation demonstrated excellent physicochemical stability, nanoscale droplet size (∼19 nm), low polydispersity, appropriate skin‐compatible pH, and significantly enhanced permeability compared with free VCZ. Notably, the combined VCZ–PSEO nanoemulsion showed superior antifungal activity against *M. canis* compared with VCZ or PSEO alone, indicating a synergistic effect. However, despite its strong performance in permeation and antifungal assays, the authors concluded that further ex vivo and clinical studies are required, particularly for its proposed application in onychomycosis [33].

In summary, while simple topical VCZ creams and gels are effective and safe for localized fungal infections, nanocarrier‐based systems (microemulsions, NLC‐gels, and nanoemulsions) consistently outperform conventional formulations in terms of skin penetration, drug retention, and antifungal efficacy. Compared with standard topical antifungals such as terbinafine, VCZ—especially when delivered via advanced vehicles—may offer faster onset of action and improved effectiveness at lower concentrations. These findings support the growing role of innovative topical VCZ formulations as promising alternatives for the management of resistant or difficult‐to‐treat superficial fungal infections.

This preliminary proof‐of‐concept study is limited by a small sample size, and the results should, therefore, be interpreted as exploratory rather than conclusive. The relatively short study duration was intentionally selected to assess early therapeutic response and lesion improvement while minimizing animal distress in accordance with ethical considerations. Extended treatment durations and longer follow‐up periods will be incorporated in future studies to further evaluate sustained efficacy and recurrence.

To strengthen the translational relevance of the present findings, future investigations should include detailed physicochemical characterization of the gel formulation, encompassing stability, drug release behavior, and skin permeation studies. Moreover, larger‐scale in vivo experiments involving additional dermatophyte spp. are necessary to substantiate the antifungal efficacy observed in this study. Comprehensive safety and tolerability evaluations, including histopathological analyses, are also required. Ultimately, these steps may pave the way for early‐phase clinical trials to evaluate the therapeutic potential and safety of topical VCZ in human subjects.

## 5. Conclusion

Results from the present study indicated that gel base VCZ 0.25% could be utilized as topical delivery for cutaneous dermatophytosis. The in vitro rate of inhibition and in vivo cure duration were markedly elevated compared with a marketed terbinafine 1% formulation. Due to the increase of drug resistance dermatophyte spp., indications of gel base VCZ for the treatment of dermatophytosis are believed to increase.

## Author Contributions

Study concept and design and technical supervision: Mahsa Fattahi and Saman Ahmad Nasrollahi; procedure: Ensieh Lotfali, Atefeh Naeimifar, Fatemeh Amiri, Ali Khamesipoor, Aliasghar Ghaderi, and Alireza Firooz; acquisition of data and drafting of the manuscript: Mahsa Fattahi, Atefeh Naeimifar, and Pegah Tamimi; critical revision of the manuscript: Shayan Zamani, Mahsa Fattahi, Saman Ahmad Nasrollahi, Aliasghar Ghaderi, and Alireza Firooz; English editing: Shayan Zamani.

## Funding

This work was supported by the Tehran University of Medical Sciences, Tehran, Iran (grant number: 99‐3‐105–50100).

## Ethics Statement

This study has been approved by the Ethics Committee of Tehran University of Medical Sciences (IR.TUMS.MEDICINE.REC.1399.1071).

## Conflicts of Interest

The authors declare no conflicts of interest.

## Data Availability

The data supporting the findings of this study are available from the corresponding author upon reasonable request.
